# Priority-setting for hospital funding of high-cost innovative drugs and therapeutics: A qualitative institutional case study

**DOI:** 10.1371/journal.pone.0300519

**Published:** 2024-03-18

**Authors:** Yasmeen Razvi, Simonne L. Horwitz, Celine Cressman, Daniel E. Wang, Randi Zlotnik Shaul, Avram Denburg

**Affiliations:** 1 Temerty Faculty of Medicine, University of Toronto, Toronto, ON, Canada; 2 SickKids Research Institute, Child Health Evaluative Sciences, Toronto, ON, Canada; 3 Department of Paediatrics, University of Toronto, The Hospital for Sick Children, Toronto, ON, Canada; 4 Department of Paediatrics, University of Toronto, Toronto, ON, Canada; 5 Department of Bioethics, The Hospital for Sick Children, Toronto, ON, Canada; 6 Department of Paediatrics, Division of Haematology/Oncology, The Hospital for Sick Children, Toronto, ON, Canada; 7 Institute for Health Policy, Management and Evaluation, University of Toronto, Toronto, ON, Canada; University of Ghana School of Pharmacy, GHANA

## Abstract

**Objectives:**

Rising costs of innovative drugs and therapeutics (D&Ts) have led to resource allocation challenges for healthcare institutions. There is limited evidence to guide priority-setting for institutional funding of high-cost D&Ts. This study sought to identify and elaborate on the substantive principles and procedures that should inform institutional funding decisions for high-cost off-formulary D&Ts through a case study of a quaternary care paediatric hospital.

**Methods:**

Semi-structured, qualitative interviews, both virtual and in-person, were conducted with institutional stakeholders (i.e. staff clinicians, senior leadership, and pharmacists) (n = 23) and two focus groups at The Hospital for Sick Children in Toronto, Canada. Participants involved in, and impacted by, high-cost off-formulary drug funding decisions were recruited through stratified, purposive sampling. Participants were approached for study involvement between July 27, 2020 and June 7, 2022. Data was analysed through reflexive thematic analysis.

**Results:**

Institutional resource allocation for high-cost D&Ts was identified as ethically challenging but critical to sustainable access to novel therapies. Important substantive principles included: 1) clinical evidence of safety and efficacy, 2) economic considerations (direct costs, opportunity costs, value for money), 3) ethical principles (social justice, professional/organizational responsibility), and 4) disease-specific considerations. Multidisciplinary deliberation was identified as an essential procedural component of decision-making. Participants identified tension between innovation and the need for evidence-based decision-making; clinician and institutional responsibilities; and value for money and social justice. Participants emphasized the role of health system-level funding allocation in alleviating the financial and moral burden of decision-making by institutions.

**Conclusions:**

This study identifies values and processes to aid in the development and implementation of institutional resource allocation frameworks for high-cost innovative D&Ts.

## Introduction

Rising costs of innovative drugs and therapeutics (D&Ts) have led to increasing challenges in allocating scarce resources for both individual healthcare institutions and broader health systems [1]. While scientific innovation has resulted in the emergence of many novel D&Ts in recent years, limited public sector resources necessitate difficult allocative decisions about which novel D&Ts to fund and for whom [1]. Particular challenges exist for paediatric and rare disease populations, as there is often limited evidence of safety and efficacy to inform decision-making, due in part to lower disease prevalence and barriers to including children in clinical research [1–3]. A clear decision-making process with explicit principles and evaluation criteria can help manage the complexity of contentious funding decisions [4–7]. For example, health technology assessment (HTA) frameworks are increasingly used in the context of resource allocation for various health interventions, including D&Ts, within publicly funded health systems [1,3,8–10]. These frameworks are employed in value-based assessments of health technologies–often premised on principles of clinical effectiveness, economic efficiency, and patient and/or societal values–to inform the allocation of limited public sector resources [3]. While some institutions have published their experiences with the development and implementation of standardized HTA frameworks, there remains a lack of published evidence with respect to allocative decision-making for innovative D&Ts within individual healthcare institutions, particularly for paediatric contexts [1,5,11–15].

In Canada, there is no national pharmacare program and drug coverage is largely regulated by provincial bodies [16]. As such, inpatient-administered therapeutics are primarily financed through provincial public sector resources. In 2020, Canadian hospital drug expenditure was approximately $4.9 billion, an increase of nearly 264% from 2001 [17]. As such, hospital formulary management is of increasing interest and concern to Canadian healthcare institutions and others within publicly funded and social insurance-based health systems internationally–especially those providing sub-specialized care to paediatric patients and those with rare diseases [18]. Such institutions are increasingly faced with requests to fund high-cost inpatient-administered D&Ts not included on hospital formularies (off-formulary), either due to their novelty or to an absence of well-established indications. Although there remains a dearth of evidence on this topic, there are a few published descriptions of institutional experiences in the development and implementation of accountability pathways or procedural approaches to formulary management, many of which are grounded in values of transparency, accountability, and consistency [5,11–13]. A coherent allocative framework based on clear substantive and procedural principles is essential to fair and sustainable institutional decision-making about funding for high-cost off-formulary D&Ts.

We sought input from institutional stakeholders in order to identify and understand the substantive principles and procedural considerations relevant to institutional funding decisions for high-cost off-formulary D&Ts at a leading academic paediatric hospital in Canada, with the broader goal of improving the quality and consistency of ethical reasoning for allocative decision-making for innovative D&Ts at comparable institutions internationally.

## Materials and methods

### Study design and setting

Our study was conducted using an exploratory qualitative design involving a series of in-depth, semi-structured, qualitative interviews with institutional stakeholders to evaluate the substantive and procedural criteria that should inform institutional decision-making for high-cost off-formulary D&Ts. It was conducted at The Hospital for Sick Children (SickKids), a quaternary care paediatric hospital located in Toronto, Canada. SickKids is Canada’s largest centre dedicated to improving children’s health through the integration of care, research, and education, providing subspecialized care to children with a variety of illnesses, including rare and orphan diseases, across the province of Ontario and beyond [19,20]. SickKids provides sub-specialized care to paediatric patients with a wide array of illness types, many of which meet established criteria for rare and orphan diseases. The current SickKids’ Off-Formulary Expensive Drug (OFED) policy was developed in 2019 and governs institutional funding for novel high-cost therapies. There is no standardized consensus on the definition of high-cost drugs, which often varies between institutions. It generally includes those D&Ts that generate exorbitant costs and/or represent a disproportionate cost relative to the total cost of the agent in terms of volume and duration [21,22]. In its current OFED policy, SickKids defines high-cost drugs and therapeutics as those costing more than CAD$50,000 annually or more than CAD$5,000 per administration. The current OFED committee consists of select standing members from senior leadership as well as rotating members based on the needs of the specific case. The committee meets to review each submission for a proposed therapeutic and provides a decision regarding its use within 72 hours. Although it articulates a process by which to adjudicate funding requests, the current policy lacks a coherent decision-making framework with clear procedural and substantive principles based on input from institutional stakeholders.

This study was approved by The Hospital for Sick Children Research Ethics Board (REB#: 1000070241).

### Data collection and analysis

Institutional stakeholders, who are staff impacted by or involved in, high-cost drug funding decisions at SickKids, were identified through institutional mapping and referral from other stakeholders through snowball sampling. Broad stakeholder involvement was sought across the institution, including from clinical departments, pharmacy, and senior leadership, using stratified, purposive sampling techniques. Potential participants were approached for study involvement through email between July 27, 2020 and June 7, 2022. Written informed consent was obtained from all participants.

Individual participant characteristics are summarized in Table 1. Approximately 30 individual stakeholders were invited to participate and 23 of them completed semi-structured interviews. The length of interviews and focus groups ranged from approximately 30 to 90 minutes. Participants were predominantly female (57%) and represented various institutional roles, including physicians in clinical roles (35%), senior hospital leadership (22%), and pharmacists (13%). Participants designated as ‘other’ did not appropriately fit under listed categories, and included a range of professions such as a registered nurse and an equity, diversity, and inclusion (EDI) expert. Two focus groups, including the institutional Bioethics Advisory Committee (n = 15) and a group of parent representatives (n = 6), were conducted.

**Table 1:** Individual participant characteristics.

	N = 23
**Gender**	**N (%)**
Female	13 (57)
Male	9 (39)
Other	1 (4)
**Institutional Role**	**N (%)**
Clinicians	8 (35)
Senior Leadership	5 (22)
Pharmacists	3 (13)
Ethicists	2 (9)
Parent advocate	1 (4)
Other	4 (17)
**Awareness & Involvement in Current OFED Policy**	**N (%)**
None	9 (39)
Minimal	6 (26)
Moderate	2 (9)
Frequent	6 (26)

OFED = Off-Formulary Expensive Drug.

Semi-structured interviews were completed either in-person at the study institution or virtually at the participant’s request. They were conducted by authors YR, SH, and DW and were recorded and transcribed verbatim. A semi-structured interview guide was used. It summarized the topics for discussion, including the principles, evidence base, and economic considerations that should guide funding decisions, as well as procedural components and transparency of the final decision-making process. The interview guide was flexible in its design and underwent iterative revisions based on emerging themes from interviews. To complement the semi-structured interviews, focus groups were conducted by YR, DW, and RZS with two institutional committees: 1) the Bioethics Advisory Committee and 2) a group of parent representatives. In order to include the full spectrum of perspectives within these committees, focus groups were felt to be most appropriate rather than individual interviews with select committee members.

Coding was conducted using NVivo 12 software (QSR Int., Ltd.) by YR and SH. Reflexive thematic analysis of the data was performed using an approach outlined by Braun and Clarke [23,24]. Initial codes were developed *a priori* based on literature review, and supplemented through the inductive development of codes during data familiarization [25]. Codes were iteratively refined throughout data collection and analysis. Emerging themes were reviewed and defined; coded data was then grouped for thematic analysis. Stakeholder interviews were conducted to expand upon emerging themes until theoretical saturation was reached. All data was anonymized and de-identified to protect participant confidentiality.

## Results

### Relevance of issue and institutional context

Participants identified resource allocation for high-cost D&Ts at the institutional level as ethically challenging but critical to explore to ensure fair and sustainable access to innovative therapies. Table 2 provides an overview of the types of funding requests for high-cost novel drugs that were approved at the case study institution between February 2021 and October 2022, retrieved from a database managed by the institution’s pharmacy department. Several participants predicted that these requests will increase in both frequency and cost, particularly with the rise of precision and gene therapies. They anticipated “a tsunami of difficult decisions”, underscoring the need for clear and robust approaches to resource allocation at the institutional level. Several participants, however, stated that the focus on institutional resource allocation for high-cost D&Ts was misplaced, contending that “setting this type of precedent is going to lead to a lot of trouble”. They argued that the responsibility for allocative decision-making about health technologies should fall to governmental health system funders, rather than individual healthcare institutions. Several participants described the current institutional policy governing resource allocation for high-cost D&Ts as *ad hoc* and lacking clear guiding principles. Furthermore, although members of senior leadership commended the existence of the current policy, they described it as overly permissive and lacking rigour in its regulatory function, raising concerns about its long-term sustainability.

**Table 2:** Summary of high-cost novel drugs approved for use at The Hospital for Sick Children between February 2021 and October 2022.

Characteristic	Value
***Patient factors***	
Age, median (IQR) years, n = 69	9 (4–14)
**Indication for IDU/OFED use, by disease area,** n = 80	
Oncology	38 (47.5%)
Immunology	22 (27.5%)
Infectious Diseases	15 (18.8%)
Cardiology	2 (2.5%)
Neurology	1 (1.25%)
Respirology	1 (1.25%)
Endocrinology	1 (1.25%)
***Drug factors***	
**Therapeutic class,** n = 80	
Antineoplastic	35 (43.8%)
Immunomodulatory agent	22 (27.5%)
Antiviral	13 (16.3%)
Haematopoietic agent	4 (5%)
Anti-asthmatic	1 (1.25%)
Antifungal	1 (1.25%)
Antilipemic	1 (1.25%)
Antiparathyroid	1 (1.25%)
Antibiotic	1 (1.25%)
***Thrombolytic*** agent	1 (1.25%)
Off-label drug use?*, n = 80	
Yes	71 (88.8%)
No	9 (11.3%)
**Nature of off-label drug use,** n = 71	
Age	42 (59.2%)
Indication	40 (56.3%)
Route of administration	3 (4.2%)
**Drugs meeting OFED criteria (exceeding $5K CAD/dose or $50K CAD/course)**	30 (37.5%)
Cost per dose, median (range)**, n = 30	$14,400 ($7150 –$83,400)
Cost per total treatment course, median (IQR)**, n = 30	$80,100 ($57,200 - $514,800)

*Indication for use, age category, or route of administration is different from Health Canada product monograph.

**Total drug cost regardless of funding source.

### Substantive principles

Substantive principles to guide resource allocation among high-cost D&Ts, as identified by participants, are described below and summarized in Table 3. Participants described key substantive criteria deemed crucial to fair and sustainable allocative decision-making, while also noting tensions between criteria and uncertainty with respect to prioritization.

**Table 3:** Substantive principles to inform institutional resource allocation for high-cost novel drugs and therapeutics.

Sub-Criteria	Selected Quotes
**Clinical evidence of safety and efficacy**	
Data from adult populations	“*[T]here’s a growing body of evidence that physiologically*, *children who are 12 and older are essentially adults*. *[…] So*, *if a child’s 12 years of age or older and the request is made for a drug in which there’s experience or even an approval in funding in the adult population*, *I would have absolutely no hesitation*.”–Clinician
Disease agnostic data	“*[I]n certain situations I would have no issue with using a drug […] where there’s some other experience that multiple diseases with the same genetic abnormality can both […] respond to that particular drug*.”–Clinician
Real-world and/or unpublished data	“*[A]nd I think that we have to be willing to accept real world data*, *whatever that is*, *and figure out a process to incorporate that into the review and approval process*.”–Clinician
Regulatory status	“*[S]omething which is approved by a regulatory authority*, *might even be approved by Health Canada for that exact indication*, *but just not on the formulary yet or reimbursement hasn’t been worked out yet*, *which will probably be the highest level of evidence*.”–Clinician
**Economic considerations**	
Direct ***financial*** considerations	“*[C]oming up with the number per dose is grossly inadequate*. *[*.* *.* *.*T]o go back to the example of SMA therapy*, *so it’s over $1*,*000*,*000*, *but there’s one treatment*, *you do it once*. *There’s another [drug] you have to give on a repetitive basis*. *So*, *you have to really look at what the total cost is over the course of therapy*.”–Clinician
Indirect financial considerations	“*[T]he challenge is that where you draw the line I guess depends on what you’re not able to do with that funding because you’re spending it on drugs instead and who’s missing out because of that*.”–Clinician
Value for money	*“How does this one stack up in terms of value for money and impact to the child*? *Because everyone always wants to use the latest and the greatest*. *And I understand that*. *But […] there is an implication for the tax*, *for the taxpayer*.*”–*Senior Leadership
**Ethical principles and values**	
Organizational responsibility	“*I think that I’ve always thought that we have an obligation to sort of try and help set the curve for how children in Canada should be treated*. *And I don’t think this issue is any different*.”–Clinician
Professional responsibility	“*[P]atients and their physician are on the same side*. *And [physicians] are advocating hard and […] it’s as disappointing to them as it is to the family*.”–Clinician
Professional responsibility–fiduciary duty	“*[W]e all trust our medical professionals*. *That’s how we show up as families*, *as patients*, *as people*. *We show up with the assumption that we have to entrust our care to our doctors*.”–Family-Centered Care Advisory Council Member
Social justice	“*There are things that need to be part of any type of program […] to make sure that we don’t miss things or we don’t create any type of inequity amongst patients and patient groups and drugs*, *et cetera*.”–Patient and Family Advocate
Patient and family values	“*You can’t disregard the patient and the patient’s family’s desires*, *their values […]*.”–Senior Leadership
Societal expectations	“*I worry more about the public*, *the reputation*. *[… A]t the end of the day*, *they can sue us*, *they may or may not be successful*, *but even if they’re not successful […] the optics of it are just terrible*, *right*?”–Senior Leadership
**Disease-specific considerations**	
Availability of alternatives	“*[T]he other the other piece of criteria that we really need to make sure that we’ve completely addressed is*, *are there other agents that are*, *have been effective in the treatment of the child’s disease*?”–Senior Leadership
Disease severity	“*[Y]ou’ve got a sense of the severity […]*. *It’s not always life or death*, *right*? *So there’s different degrees of […] what is this therapeutic going to do for the child and how does that factor in alongside the various perspectives*.”–Senior Leadership
Prioritization of need	“*So if you’re going to be blind*, *but now you’re going to be 20% visually impaired*, *is that more important than the person who has […] that round the clock ventilator support that’s only going to need overnight ventilator support*? *I don’t know which one of those things is more important […]*. *And yet*, *if you want to actually resource allocate*, *you’re going to have to come up with a plan*.”–EDI Advocate

### Clinical evidence

Participants consistently highlighted the importance of using clinical evidence of safety and efficacy to support allocative decision-making; it was frequently described as the most salient substantive criterion. Specifically, participants recommended considering anticipated treatment-related reductions in morbidity and mortality, as well as a given therapy’s safety profile, as key inputs to allocative decision-making.

While participants emphasized the importance of evidence for a given intervention, specifying a minimum level of acceptable evidence to inform decision-making presented a challenge. Participants frequently noted the challenges associated with meeting established evidentiary requirements, such as phase III clinical trial data, given the rarity of many of the conditions for which novel D&Ts are indicated. Most participants argued that children are inherently disadvantaged due to the limited use and evaluation of certain D&Ts in paediatric populations, most notably for rare diseases.

“Adults with lung cancer […], there’s enough of those patients to be able to study […] and there’s enough data for them to get approved and they get funded therapy, but a child who’s got a rare cancer where we want that same drug can’t get [it].”–Clinician

Participants frequently stated that they would therefore feel justified extrapolating data that was disease agnostic and specific to adult populations or that was taken from unpublished and/or real-world sources. The role of innovation as a substantive principle was also discussed in the context of generating and appraising evidence for novel D&Ts in the paediatric population, with many participants stating that innovative drug use was critical to advance the literature on emerging therapeutics. Looking to data from previously approved cases in evaluating a given D&T was also discussed; the use of past precedents in ensuring consistency in the allocative decision-making process is discussed further under ‘Procedural Considerations’. Federal regulatory approval was also suggested as an evidentiary benchmark for clinical safety and efficacy, with the highest standard of evidence attributed to D&Ts already approved by Health Canada or a foreign stringent regulatory authority, even if approved only for a different population or alternative indication. Participants were also willing to accept lower levels of evidence for patients with more severe disease states who had failed previous lines of established therapy.

“[W]hen you deal with patients in relapse situations where [the] standard of care has failed, then I think it’s not reasonable to apply the same standard and say, no, we want a New England Journal or [top-tier journal] paper […] otherwise we won’t even consider it.”–Clinician

### Economic considerations

Participants highlighted economic considerations as another key substantive criterion. Although rarely considered to be the most important criterion, cost constraints were often acknowledged to be the ultimate limiting factor in approving funding requests. The degree to which participants valued economic considerations varied based on their institutional roles, with members of senior leadership placing greater importance on economic considerations compared to clinicians and patient and family advocates. While stringent economic limitations were suggested theoretically, participants anticipated placing less importance on such constraints when considering individual requests, and instead prioritizing other substantive criteria. Key economic considerations included direct financial considerations, consideration of opportunity costs, and notions of value for money and economic efficiency. Direct financial considerations were understood to encompass both the upfront monetary cost of a therapy and the cost of future administrations, as well as the availability of alternative funding streams to reduce institutional costs, such as pharmaceutical companies’ compassionate access programs. Participants referred to opportunity costs within the overall budget, specifically the institution’s ability to continue to fund other critical services and infrastructure. Value for money was also suggested as a substantive criterion to include, although difficulties in both defining and assessing value were highlighted, and numerous ethical quandaries raised with respect to the inherent value judgements associated with this concept.

### Ethical and social values

Participants proposed numerous ethical and social values for consideration in allocative decision-making, including organizational responsibility, professional responsibility, social justice principles, and societal expectations. Organizational responsibility encompassed the institution’s responsibility to the broader paediatric population and overlapped with considerations of opportunity costs. Organizational responsibility also related strongly to commitments to innovation. Given that academic paediatric centres are largely responsible for the generation of clinical data on novel D&Ts, participants expressed that hospitals have a responsibility to invest in these therapeutics to advance innovation.

“Because if it’s something for children, if we don’t do it here [at a quaternary paediatric institution], then who’s going to do it […]? […Doing it] would be a service not only to our patients, but also […] to the larger paediatric community.”–Clinician

Patient and family advocates stated that the organizational culture should promote the use of exploratory treatments for patients who have not responded to multiple lines of therapy. While clinicians similarly recognized the importance of innovation, many emphasized the importance of alternative funding mechanisms where clinical evidence is limited or lacking, including through formal clinical research endeavours, such as single-patient studies sponsored by pharmaceutical manufacturers.

Professional responsibility centred largely on a physician’s fiduciary duty to her individual patient in pursuit of a given therapeutic, which was seen to both include, and at times conflict with, a commitment to the practice of evidence-based medicine and stewardship of public resources. Patient and family advocates placed particular weight on fiduciary duty; many stated that if a clinician proposed a high-cost therapeutic, they would trust that the recommendation was based on a careful calculus of its relative benefits and harms. Patient and family representatives also expressed that clinicians’ professional duty includes an obligation to seek out and exhaust all reasonable therapeutic options, for each affected child as well as in pursuit of innovation.

Participants frequently invoked social justice principles, including equity, diversity, inclusion, and distributive justice. Concerns surrounding inequities in access to information about the availability and relevance of innovative D&Ts, as well as the ability of families to navigate the health system to advocate for their children, were highlighted, especially with respect to marginalized populations. Patient and family representatives raised concerns that unconscious biases of clinicians may impact their propensity to assist particular patients in accessing the allocative decision-making process. Participants also noted that families with greater access to social resources and awareness of available health services may similarly have greater ability to access novel D&Ts.

“[Y]ou’ve always got families that are squeaky wheels, and oftentimes the squeakiest wheels can get sort of expedited to the top of the list because they’re there and they’re advocating. [… H]ow do these processes work for racialized and marginalized peoples?”–Senior Leadership

Some participants argued that clinicians have a responsibility to reduce these inequities by recognizing their own implicit biases and by educating patients on available interventions, various funding avenues, and emerging D&Ts. Participants stated that a clinician’s role includes advocating for each patient within this overall process. Substantial overlap was highlighted between the notion of distributive justice and opportunity costs.

“I also still struggle with the fact that if we’re able to pay $2,000,000 for this one patient […] why are we not paying $2,000,000 to make sure that all Indigenous children have access to even just primary care every single day?”–Equity, Diversity, and Inclusion (EDI) Advocate

Finally, participants contended that societal expectations should be considered, albeit to a lesser degree than other identified substantive criteria. Although participants believed that the risk of litigation against the institution was low, they nevertheless voiced concerns about funding decisions causing potential damage to patient and family relationships and eroding public trust. Participants noted that the public perceives paediatric institutions as providers of the highest level of innovative care for children, but worried that this belief may be detrimental. There was concern that this perception may lead to unrealistic individual and societal expectations of what an institution can do for a given child. This perception underscored the urgency to implement a clear policy in order to make defensible D&T funding decisions and better protect the institution’s reputation.

### Disease-specific considerations

Participants highlighted disease-specific considerations as valuable substantive criteria to inform allocative decision-making. Disease-specific considerations encompassed disease severity as well as evidence and availability of established alternatives. Participants stated that patients should be considered refractory to established treatment prior to seeking out novel therapies through this process. They also took into account existing comorbidities and disease progression, which could impact the success of a proposed therapeutic. The potential impact of early therapeutic intervention on developmental outcomes was not discussed by participants, with much of the focus instead centering on acute impacts to physical health. Participants with clinical roles noted the importance of taking into account the limitations of medical interventions and the potential to prolong suffering, necessitating balancing the patient’s clinical condition and ability to withstand treatment with the desire to exhaust all possible treatment options.

“It has to be seen in the context of what’s the historical outcome, the current outcome, and what is sort of the potential best-case scenario? And where in the trajectory is the patient, the family that we’re dealing with here right now?”–Clinician

### Procedural considerations

Key procedural components of a just and effective allocative decision-making process, as identified by participants, are described below. Overall, compared to the substantive criteria identified, there was greater uncertainty cited by participants with respect to the ideal procedure that a funding policy should follow.

### Stakeholders

Participants identified several institutional stakeholders, reflecting diverse professional and social backgrounds, as critical to allocative decision-making processes. Important qualities of involved stakeholders included relevant clinical expertise, lack of conflicts of interest, capacity for objectivity, advocacy skills, strong working relationships with peers, and a commitment to serving on the decision-making committee for a sustained period. Participants underscored the importance of including stakeholders with expertise in equity and social justice principles and recommended that stakeholders undergo implicit bias training prior to participation in the decision-making process.

Maintaining a rotating group of committee members, but including select standing members, was deemed to be important in ensuring an efficient and reliable decision-making process. The relevant stakeholders named by participants included senior leadership, clinicians, pharmacists, social workers, and ethicists. Participants raised concerns regarding the involvement of senior leadership, particularly by patient and family representatives, who suggested that this could introduce a hierarchy among decision-makers and lead to decisions driven primarily by budgetary considerations. The inclusion of senior leadership, however, was described by most to be necessary, given the potentially significant financial implications for their institutions. Participants also highlighted pharmacists as integral stakeholders given their specialized knowledge of appropriate dosing, drug indications and interactions, procurement channels, and alternative financing options. A drug access navigator–a dedicated pharmacist with specialized expertise on drug acquisition and funding pathways for novel D&Ts–was suggested as a valuable resource for clinical teams submitting funding requests and navigating this process. Participants suggested involving external consultants, such as equivalent stakeholders at other paediatric institutions, in reviewing funding decisions to ensure neutrality, although concerns surrounding confidentiality and feasibility were also raised. Generally, participants agreed that there should be no hierarchy amongst decision-makers and that no added weight should be given to any single stakeholder’s views.

Participants expressed varied opinions on the role of patient and family representatives. They highlighted the importance of including the patient or family perspective and acknowledged the potential benefit of including families in decision-making to better understand their values. Simultaneously, however, participants acknowledged several associated challenges. For example, questions arose about whether the affected family should be involved in decision-making for their child or, rather, whether there should be a general patient advocacy group or representative. Concerns were specifically raised about potential conflicts of interest and the ability of families to maintain unbiased positions.

“I should be saying yes, families and patients involved at every step of the way […] but it’s just not possible. It’s just not possible for them to remain objective. It’s not possible for them to advise on access to a medicine without the sort of very in-depth knowledge of all that that would entail.”–Patient and Family Advocate

Patient and family representatives interviewed in the focus groups, however, described feeling that their involvement was paramount to defensible and transparent decision-making and that they would be well-equipped to make rational decisions in the best interests of their children.

### Structure of the allocative decision-making process

As a starting point, participants agreed that there should be an established cost threshold to trigger the allocative decision-making process, although the precise threshold was debated. Some participants raised the possibility of tiered thresholds, to enable variations in the adjudication process based on the cost of the therapy under review, such as increasing involvement of senior hospital leadership in decision-making at higher cost thresholds. The urgent nature of these funding decisions was repeatedly highlighted by participants, underscoring the importance of an efficient turnaround time for decisions.

Participants also recognized that the institutional allocative decision-making process is not necessarily the appropriate avenue for all funding requests for innovative D&Ts, particularly those with limited clinical evidence on which to premise requests for use, which may therefore be better suited to access through research pathways. In tandem with the institutional process, participants expressed that partnerships with relevant government agencies and pharmaceutical companies should be strengthened to share in the burdens of both evidence generation and exceptional financing.

“[T]he first decision point is again, is there enough evidence to warrant that this drug is a completely appropriate therapy and if not, then it goes down a completely different pathway […]. It goes down a research pathway. If there’s evidence, we can still seek […] other external sources of funding, such as compassionate use or company programs.”–Clinician

Once a funding request is deemed appropriate for institutional funding consideration, multidisciplinary deliberation, ideally where stakeholders are blind to the submitting clinician and patient, was identified as an essential procedural component of defensible decision-making. Participants suggested that the process conclude with either a majority vote or a consensus-based decision. A consensus was acknowledged as ideal, although potentially impractical, with concerns of potential attenuation of opinions due to coercion or the desire for conformity.

“[…T]he ideal state would be a really nimble Supreme Court. […Y]ou make your case, you bring all your evidence, and then you have a group of stakeholders with experience and knowledge in the area who can turn around, ask questions, and then give a well thought out, rational decision.”–Clinician

The use of past decisions as precedents upon which to base future decision-making was discussed by participants. Ensuring consistency in both the process and outcomes was noted to be important for fostering public trust and confidence in the process. Participants acknowledged, however, that consistency in outcomes would come at the expense of flexibility and the ability to consider a patient’s unique clinical and social circumstances within the decision-making process. It was therefore acknowledged that the rarity of these conditions may necessitate certain case-by-case considerations, as is done within the current institutional policy, rather than a rigid outcomes-based framework based solely on precedents, highlighting a tension in the desired structure of the revised policy.

“[Y]ou’re trying to create something that people can rely on in the myriad of circumstances. And just when you think [of] all of the variations in the themes in terms of how things might unfold or what factors might be at play, then there’s something else. [… Y]ou don’t want to be changing the policy constantly […] every time there’s a new case.”–Senior Leadership

Participants also recommended incorporating a process to proactively evaluate anticipated funding requests for emerging therapeutics. Such a process was suggested in order to allow for greater opportunity to consider and deliberate on a novel therapeutic, without the typical time constraints associated with urgent allocative funding requests.

“[W]e should start to […] think about this drug before we get the request, because we’re often scrambling a little bit. […] There’s always a sense of urgency that this child’s life is on the line here. So, I think if we can be a bit more proactive, it’ll serve us well.”–Senior Leadership

### Implementation

The manner in which decisions should be communicated and to whom was debated by participants. There was unanimous agreement that final, written decisions should be communicated by the committee to the most responsible physician and the patient and family, along with the rationale for either accepting or denying the request. Participants deemed sensitive communication of decisions to be especially important for marginalized populations, particularly in circumstances where a given request was denied. For transparency, some participants suggested that these decisions should be communicated to the relevant clinical departments. Participants discussed the possibility of reporting aggregate data on innovative drug funding requests on an annual or semi-regular basis. However, participants were concerned that given the rarity of these cases, any clinical information, even if anonymized, may lead to the identification of the patient, compromising confidentiality. Participants acknowledged, but were unable to resolve, this tension between the desire for transparency with the need to preserve patient confidentiality, with one clinician participant noting that: “transparency is a core value […]. The problem is, you can’t disclose that stuff really publicly or even broadly internally without violating patient confidence […].”

Participants also emphasized the importance of data tracking and decision evaluation. Assessing the efficacy of innovative use of a therapeutic was described as an important step in contributing to its body of evidence, with corollary implications for how future funding decisions around that therapeutic are approached. The evaluation of clinical efficacy per pre-established endpoints would also help determine the rationale for, and legitimacy of, ongoing funding to support continued administration for the patient in question. Evaluating potential inequities in decision-making based on sociodemographic factors including race and income was also highlighted as an important feature of evaluating this process.

Finally, the importance of an appeals process was endorsed by the majority of participants, who characterized it as fundamental to good governance. Participants were divided with respect to the ideal operation of an appeals process, with some participants describing the need to convene a new group of stakeholders to review the request, while others suggested that the original group of reviewers could objectively consider an appeal in the context of new information. The latter was generally described as more feasible when accounting for the urgency to review an appeal and make a decision within a short timeframe. Participants also questioned whether a statute of limitations should apply or whether it would instead be reasonable to consider new evidence to support an appeal at any point upon the discovery of new information.

### Emerging tensions amongst substantive and procedural considerations

Participants identified a number of tensions between substantive and procedural considerations, summarized in Table 4. Innovation, described by participants as the novel use of therapeutics with a minimal and/or emerging evidence base, conflicted with a range of other substantive principles, including the need for clinical evidence of safety and efficacy. While supporting evidence was seen as a requirement for justifiable resource allocation, participants emphasized the potential value of this decision-making process in pursuing innovative use of novel therapeutics with a limited body of evidence. An additional tension surfaced between social justice principles and the desire to generate clinical evidence in populations who have historically had limited opportunities for participation in clinical research, notably racialized populations. Participants acknowledged that families facing marginalization may perceive recommendations of therapeutics with limited evidence of efficacy as experimentation, given the history of medical violence against such communities and ongoing mistrust of the healthcare system. Similarly, tension emerged between innovation and distributive justice, founded on concerns that academic institutions have a greater propensity to invest in medical and technological innovations than in initiatives addressing social determinants of health.

**Table 4:** Emerging tensions among select substantive and procedural criteria.

Tensions	Selected Quotes
Desire for innovation vs. need for clinical evidence	“*The other threshold [for denial] is if there’s absolutely a complete absence of supporting evidence*. *But that’s even a relative one […] like if you said no to that*, *then nothing would ever get discovered*.”–Senior Leadership
Technological innovation vs. social justice	“*[Approaching a family with low-evidence for a D&T] could trigger some trauma*, *and it could trigger*, *you know*, *that continued mistrust of the system*, *and then they may also then feel like*, *oh you want us to be guinea pigs*.”–EDI Advocate
Investment in innovation vs. distributive justice	“*[W]e’re much more interested as a society in spending a great deal of money on […] rescue with enzymes or cellular therapy […] than we are to actually spend money on the minoritized*, *racialized populations in the GTA who might benefit from stable housing […] or children getting lunches*.”–EDI Advocate
Value for money vs. social justice	“*[V]alue for money is always really hard [be]cause the very name of it is a value judgment*. *[… I]t’s normative*, *and so you’re taking normal to be this ablest view of the world and then counting your number of points to as close to normal or far from normal*.”–EDI Advocate
Patient/family involvement vs. social justice	“*[E]nsuring that you have families that are on this type of decision-making committee only further adds to the inequities I think that you’ll see that is out there because those people that have the time or ability to sit on these types of committees are traditionally the people that already are in those more privileged positions*. *[…] And so they’ll end up sort of representing the views and values of their own position and families that are like them rather than the patient community as a whole*.”–Patient and Family Advocate
Clinician vs. institutional responsibilities	“*[W]e do have a responsibility to the entire single-payer healthcare system*, *that’s clearly there*, *but I think most practitioners will agree with me that we have an even bigger responsibility to give a particular child the best available care*.”–Clinician

The concept of value for money was found to conflict with social justice principles, as there was concern that definitions of value would be based on normative and ableist views. Participants also raised concerns regarding the involvement of patient and family stakeholders in allocative decision-making processes, specifically, that it may exacerbate existing inequities in access to care as families experiencing greater levels of privilege were presumed to have the social resources needed to better advocate for their child within this process. An inherent tension was also identified between the fiduciary duty of clinicians to their individual patients and institutional budgetary considerations. While most clinicians recognized that they should be keeping the broader budget and associated opportunity costs in mind, they reported feeling that this was overridden by their duty to their individual patient.

## Discussion

The results of the present study highlight key substantive and procedural criteria to be considered for institutional allocative funding decisions for high-cost inpatient-administered D&Ts. Substantive principles deemed integral for just and sustainable resource allocation included clinical evidence of safety and efficacy; economic considerations; ethical principles including social justice, professional and organizational responsibility, and societal expectations; and disease-specific considerations. Participants raised concerns surrounding the paucity of robust evidence of safety and efficacy to support funding decisions within specialized populations such as paediatrics. This finding is generally consistent with existing literature, which encourages the use of real-world evidence in the face of limited clinical trial data, including the identification of existing and/or retrospective data [26,27]. This suggests that the tension between the need for clinical evidence of safety and efficacy and the desire for innovation in available therapeutics may be diminished by utilizing a decision-making process that incorporates the identification and assessment of real-world data, both in support of a given funding decision and as a means of advancing the available evidence, as a number of HTA institutions internationally are exploring.^4,5,18,28–30^ While the need for greater clinical evidence to support this decision-making process is well-documented in the literature, its relative importance varies amongst Canadian institutions.^18^ Pharmacy directors and members of institutional D&T committees at ten Ontario hospital corporations were surveyed and rated the quality of clinical evidence to support new formulary drug requests as being more favourable than their economic evidence. This may, however, reflect the rarity of conditions and novelty of requested therapeutics at the case study institution relative to these surveyed hospitals.

The importance of incorporating ethical principles into allocative decision-making was also emphasized by participants, particularly with respect to social justice principles of equity, diversity, inclusion, and distributive justice. The increasing acknowledgement of social justice within healthcare funding decisions is well-documented, with examples including the UK’s National Institute for Health and Care Excellence granting priority to historically disadvantaged populations within HTA funding decisions, as well as several institutions emphasizing the importance of equity of access [28–34]. Overall, the relative prioritization of the described substantive principles presented a challenge, a finding that is substantiated by the current literature on institutional resource allocation. This suggests that while having a clear hierarchy of values may relieve the moral and ethical distress within such decision-making, the codification and relatively equal weighting of these values may instead be the optimal path forward.

Economic considerations identified by participants include direct costs of treatment, opportunity costs to the organization, and value for money. Available literature has similarly discussed both the cost of treatment to the organization and the patient, as well as impacts on the ability to fund other critical services or infrastructure within a given institution [4,5,18,33,35,36]. Furthermore, cost-effectiveness considerations remain a key criterion within institutional allocative decision-making, although there is little available guidance on how to best conduct such analyses. Participants also described uncertainty about the ideal procedural components to ensure a just and effective allocative decision-making process, specifically related to cost thresholds. From the outset, participants were unable to commit to a specific cost threshold necessary to trigger the allocative decision-making process, but suggested implementing a number of parallel processes that incorporate higher levels of scrutiny for higher-cost therapeutics. In the face of increasingly prohibitive drug costs, participants also described a strong idealistic desire to have the cost of drug prices regulated and controlled. To help address this, there is a clear role for future advocacy to government agencies and pharmaceutical companies, both with respect to the actual cost of available D&Ts as well as the introduction of additional avenues for funding of novel therapeutics that are insufficiently served by system-level HTA processes.

Consistent with prior literature focused on procedural justice in health system priority-setting, participants highlighted multi-disciplinary deliberation ending in either a consensus or majority vote as a way to encourage effective stakeholder participation in decision-making and promote the expression of differing views while guarding against undue influence [37,38]; this was identified as an essential procedural approach to defensible decision-making. In similar work on institutional priority setting in a Canadian context, the potential for power imbalance between stakeholders has also been described as a limiting factor in the allocative-decision making process [37]. To remedy this, an “empowerment condition” has been proposed, which outlines the need for efforts to minimize such power differences within priority setting and allow for diverse participation in this process. Although seeking a breadth of stakeholder perspectives was considered ideal among participants, there were concerns about this leading to decision-making delays, a concern corroborated by prior literature [30,39,40]. There was also disagreement about the most appropriate stakeholders to include in the process, with the role of patients and families most contested, largely due to concerns of potential conflicts of interest. Furthermore, participants emphasized the importance of data tracking and evaluation of clinical outcomes and equity after a given decision is made. The integration of formal health equity assessments, as has been conducted within some paediatric institutions, may be used reflexively in the implementation of a resource allocation framework [41].

The tension between transparency and confidentiality underscored participants’ discomfort with how to best communicate the results of funding decisions. Although ideal to disseminate anonymized results of decisions within the institution in a timely manner, the rare nature of the funding requests necessitates aggregating a relatively large amount of data, perhaps spanning multiple years, in order to best maintain patient confidentiality.

### Implications for future policy and research

This study underscores a well-described health system gap in novel D&T funding–especially for paediatric and rare diseases–that is currently being filled by *ad hoc* committees and policies at individual healthcare institutions, including academic paediatric centres. To ensure fair and sustainable population access to innovative technologies, there is a clear need to develop innovative mechanisms of adjudication and funding for novel D&Ts by public health system payers. Work has already been undertaken to innovate and contextualize values-based assessments for resource allocation in paediatric and rare disease contexts through existing HTA frameworks [1,3,14,15,42,43]. The implementation of local decision-making policies will likely remain necessary, as institutions continue to face resource allocation challenges for high-cost innovative D&Ts, which are unlikely to be resolved entirely through reform of system-level HTA paradigms or appeal to governmental payers.

The goal of our study was to identify key substantive and procedural principles, and describe associated tensions, integral to include in resource allocation frameworks in order to ensure ethical and sustainable resource allocation decision-making processes for high-cost D&Ts at individual healthcare institutions. The creation of a revised evidence- and values-informed institutional policy drawn from a broad range of stakeholder perspectives is required in order to further advance this work. There are, however, clear challenges with practically creating a framework that resolves the inherent tensions and is able to evaluate evidence, complete a cost-benefit analysis, address concerns of equity and inclusion, incorporate an effective evaluation framework, and follow a fair and equitable procedure. Although these challenges were well-described by stakeholders, implementing structured yet flexible institutional decision-making frameworks is necessary to manage the complexities of difficult funding decisions and to share the associated moral burden. Despite the described challenges, the present work has made important progress in elucidating key values and processes to support evidence-informed recommendations to reform existing institutional policies for high-cost innovative drugs.

Our study considered only inpatient-administered D&Ts and does not take into account sources of funding for ongoing outpatient therapies or adjunct therapies; this is an important area of future study. Furthermore, the role of patients and families in funding decisions, a significant point of contention, must be further explored. Future work should elucidate both the extent and ideal type of their involvement, as well as ways to ensure that families with limited social resources have similar opportunities for participation.

### Limitations

Results from the present study were derived from qualitative interviews conducted within a single institution in a large Canadian city. This may therefore limit the transferability of these findings to other academic healthcare institutions, paediatric or otherwise, particularly those operating within privatized healthcare systems or other cultural contexts. Similarly, the patient and family representatives surveyed in the focus groups were not necessarily representative of the range of sociodemographic perspectives at play in a multicultural polity like Canada; marginalized populations, in particular, may have been under-represented. As a result, the themes uncovered from the present study may not be representative of all stakeholders impacted by, and involved in, the funding of high-cost D&Ts. Finally, participant responses may have been influenced by recall bias and/or social desirability bias.

## Conclusions

The present study details critical values and ideal processes to aid decision-makers in implementing a fair and consistent approach to institutional resource allocation for high-cost, innovative, inpatient-administered D&Ts. The relative prioritization of substantive principles within institutional resource allocation remains a significant challenge, but may signal that consideration of unweighted, rather than prioritized, values is the appropriate path forward. These results will aid in the formation of an evidence- and values-informed framework for institutional resource allocation decisions for high-cost novel D&Ts. Future work should aim to elucidate the optimal role of patients and families within this decision-making process, as well as the role of government health system funding in alleviating the financial and moral burden of this process from individual institutions.
